# New Hydronephrosis in the Native Kidney Is Associated with the Development of De Novo Urinary Bladder Urothelial Carcinoma in Patients with Post-Kidney Transplantation

**DOI:** 10.3390/healthcare11091209

**Published:** 2023-04-23

**Authors:** Cheng-Ju Ho, Yu-Hui Huang, Tzuo-Yi Hsieh, Min-Hsin Yang, Shao-Chuan Wang, Wen-Jung Chen, Wen-Wei Sung, Sung-Lang Chen

**Affiliations:** 1 Department of Urology, Chung Shan Medical University Hospital, Taichung 402, Taiwan; cshy1711@csh.org.tw (C.-J.H.); cshy637@csh.org.tw (T.-Y.H.); cshy1543@csh.org.tw (M.-H.Y.); cshy764@csh.org.tw (S.-C.W.); cshy1210@csh.org.tw (W.-J.C.); flutewayne@gmail.com (W.-W.S.); 2 Institute of Medicine, Chung Shan Medical University, Taichung 402, Taiwan; 3 Department of Physical Medicine and Rehabilitation, Chung Shan Medical University Hospital, Taichung 402, Taiwan; yhhuang59@hotmail.com; 4 School of Medicine, Chung Shan Medical University, Taichung 402, Taiwan

**Keywords:** kidney transplantation, hydronephrosis, urinary bladder, urothelial carcinoma

## Abstract

Increased malignancy after kidney transplantation (KT) is by far the most troublesome issue. Among these malignancies, urothelial carcinoma (UC) incidence is uniquely high in Taiwan. We want to know whether routine sonography to detect native hydronephrosis is associated with the development of de novo urinary bladder urothelial carcinoma (UBUC) in post-KT recipients. From 2003 to 2018, we retrospectively analyzed 1005 KT patients, 58 of whom were subsequently diagnosed with UBUC. The association between new native hydronephrosis and post-KT UBUC was analyzed with univariate and multivariate logistic regression analyses and a Kaplan–Meier plot. We excluded cases of people who had upper urinary tract urothelial carcinoma (UTUC) and were diagnosed prior to UBUC. There were 612 males (60.9%) and 393 females (39.1%), with a mean age of 48.2 ± 12.0 years old at KT. The mean follow-up period was 118.6 ± 70.2 months, and the diagnosis of UBUC from KT to UBUC was 7.0 ± 5.1 years. New native kidney hydronephrosis occurred more frequently in the UBUC group (56.4% versus 6.4%, *p* < 0.001) than the non-UBUC group. Multivariate analysis disclosed that native hydronephrosis is the only statistically significant factor for UBUC, with an odds ratio of 16.03 (95% CI, 8.66–29.68; *p* < 0.001). UBUC in post-KT patients with native hydronephrosis also showed a tendency toward multifocal lesions upon presentation (47.8%). Post-KT UBUC is characterized by pathologically aggressive and multiple foci lesions. Native kidney hydronephrosis may be a deciding factor of post-KT UBUC.

## 1. Introduction

Kidney transplantation (KT) is the treatment of choice for a minority of patients with end-stage renal disease (ESRD). Marked improvements in early graft survival and long-term graft function have made KT a more cost-effective alternative to dialysis. The outcomes of KT improvement may be attributed to the introduction of highly active immunosuppressive agents in recent decades. Despite improvements in graft and patient survival and reductions in acute rejection rates after newly immunosuppressive agent induction, de novo malignancy after KT remains one of the lingering causes of mortality for KT recipients [1,2].

Overall, compared with the general population, a two- to seven-fold elevated risk of malignancies was documented among KT recipients [3]. Nonmelanoma skin cancer and lymphoproliferative disorder are frequent cancers in Western countries; however, a previous study reported a remarkably increasing incidence rate of urothelial carcinoma (UC) [4]. Compared with European recipients, Asian KT recipients face a higher risk of developing de novo bladder cancer [1]. For UC caused by KT in Taiwan, previous studies have extensively reported an incidence rate of approximately 3.5–4.1% [2,5,6]. The custom of using Chinese herbs and the intake of arsenic from underground water in Taiwan may contribute to the development of post-KT UC.

Urinary bladder urothelial carcinoma (UBUC) is a common cancer worldwide, ranking as the 9th leading position. There are near 549,000 new cases and 200,000 mortalities in 2018 [7]. Generally speaking, non-muscle invasive bladder carcinoma (NMIBC) leads to a relatively high 5-year survival rate (90%); those that progress to muscle invasive bladder carcinoma (MIBC) have a decreased 5-year survival rate (approximately 70%). Moreover, the 5-year survival rate in patients with metastatic UBUC is only a miserable 5~35% [8]. However, the heterogeneous and unpredictable characteristics of UBUC make diagnosis a hard nut to crack. The same tumor stage may present with disparate clinical outcomes in different individuals.

Until now, there have been several tissue-based biomarkers, such as bladder tumor antigen (BTA) and nuclear matrix protein 22 (NMP 22), which have been tested for their prognostic roles in UBUC, but none of those, so far, can be used for the early and precisely noninvasive detection of UBUC [9]. The unmet needs preclude their extensive use in routine clinical practice [10]. Therefore, the early detection of UBUC in post-KT patients is crucial for improving oncologic outcomes. Except for established risk factors, such as smoking and polycyclic aromatic hydrocarbons, BK virus and human papillomavirus can also cause UBUC oncogenesis, which can be detected using renal biopsy, urine cytology, or blood analysis [11,12]. Periodic renal sonography for native and graft kidneys is usually the routine follow-up protocol for KT patients. Native hydronephrosis is a risk factor for developing upper tract urothelial carcinoma (UTUC) and UBUC in hemodialysis patients [13]. Furthermore, our previous study demonstrated an association between native hydronephrosis and UTUC in post-KT recipients [14]. However, the role of native hydronephrosis in de novo UBUC in post-KT recipients has not yet been well elucidated.

Therefore, we conducted this study to investigate whether native hydronephrosis is a predictor of the development of de novo UBUC in post-KT recipients.

## 2. Materials and Methods

This retrospective cohort study was approved by the Institutional Review Board of Chung Shan Medical University Hospital, Taichung City, Taiwan (IRB number: CS2-23019). From January 2003 to September 2018, we enrolled cases of patients with KT who were over 18 years old and who had regular medical visit records or had discontinued follow-up for a known reason. Those who had malignancy before KT or UTUC diagnosed prior to UBUC were excluded from our analysis. We retrospectively analyzed 1005 KT patients, 58 of whom were subsequently diagnosed with UBUC within the study period. The immunosuppressive agents used post-KT included calcineurin inhibitor (cyclosporine or tacrolimus), mycophenolate mofetil or azathioprine, and steroids without CD3 monoclonal antibodies or anti-thymocyte globulin induction therapy. The follow-up protocol after KT at our institution included blood examination, urine analysis, and monthly urine cytology for six months and then quarterly thereafter. Renal ultrasonography was essential every three months during the first year and then biannually. New hydronephrosis in a native or graft kidney was defined based on ultrasonography or computed tomography (CT) image interpretation or a documented report in the medical records. Unilateral and bilateral native hydronephrosis were unitedly categorized into the hydronephrosis group. Patients with evidence of hydronephrosis before KT were also removed from the post–KT hydronephrosis group. As long as patients presented with hematuria or hydronephrosis in their native kidneys were noted during follow up, cystoscopy with bilateral retrograde pyelography or ureterorenoscopy (URS) were undertaken. Urine cytology was collected via voiding or URS flushing. In the setting of positive urine cytology without visible bladder lesions, random biopsy is performed based on urologist discretion. An abdominal CT scan was performed when the findings from the aforementioned procedures were abnormal or inconclusive. Patient’s characteristics, including smoking and hypertension histories, were retrieved from documented medical records. Other patient co-morbidities, such as diabetes, dyslipidemia, hyperuricemia, hepatitis B virus (HBV), hepatitis C virus (HCV), and BK virus infection diagnosis, were determined based on biochemistry and serological tests. UBUC pathological diagnosis was confirmed using a peer-reviewed examination by senior and experienced pathologists and specimens were obtained via previous cystoscopic biopsy, transurethral resection of bladder tumor, or cystectomy. 

A chi-square test and an independent sample *t*-test were used to compare categorical variables. Univariate and multivariable logistic regression models were fitted to examine the associations of UBUC. The association between patients with and without native hydronephrosis and time to UBUC was estimated using the Kaplan–Meier method and a log-rank test. All statistical tests were based on a two-sided significance level of 0.05, with *p* < 0.05 indicating statistical significance. SPSS statistical software (version 15.0; SPSS, Inc., Chicago, IL, USA) was used for all statistical analyses. 

## 3. Results

During the study period, which spanned more than 15 years, a total of 1005 KT recipients followed up at our hospital, enrolling 612 males (60.9%) and 393 females (39.1%), with a mean age of 48.2 ± 12.0 years at KT (Table 1). The mean follow-up interval was 118.6 ± 70.2 months, and 47.1% of patients had received over the 10-year follow-up period. Of the enrolled subjects, 22.7% has a smoking history. Common systemic diseases such as hypertension, diabetes mellitus, dyslipidemia, and hyperuricemia had a prevalence of over 50%. A relative high prevalence of HBV and HCV infections was noted in 31.3% and 19.1% of patients, respectively, and the BK virus infection rate was about 39%. There were 88 (9.4%) patients with native hydronephrosis during the study follow-up period, and most of them were unilateral (74/88, 84.1%). The mean time from KT to UBUC diagnosis was 7.0 ± 5.1 years.

The characteristics of KT patients with and without UBUC are demonstrated in Table 2. At the end of the study, 58 patients were diagnosed with UBUC, which was confirmed by pathological specimens. The age at KT was higher in patients with UBUC compared with the non-UBUC group (51.6 ± 9.5 versus 48.0 ± 12.0, years, *p* = 0.029), and the UBUC group predominantly contained females (63.8% females versus 36.2% males, *p* < 0.001). Furthermore, statistically significant lower smoking rates were observed in the UBUC group (12.2% versus 23.5%, *p* = 0.025). There were no significant differences in systemic diseases, including hypertension, diabetes mellitus, dyslipidemia, and hyperuricemia, and similar trends were observed for viral infections between the two groups. Compared with the non-UBUC group, there were 31/58 (56.4%) patients in the UBUC group who had native hydronephrosis. Conversely, only 57/947 (6.4%) patients had native hydronephrosis in the non-UBUC group, *p* value < 0.001. Moreover, 29/31 (93.5%) patients in the UBUC group had unilateral native hydronephrosis, compared with 45/57 (78.9%) patients in the non-UBUC group. However, graft hydronephrosis was not significantly different in post-KT patients with or without UBUC groups (21.8% versus 23.2%, *p* = 0.811).

**Figure 1:**
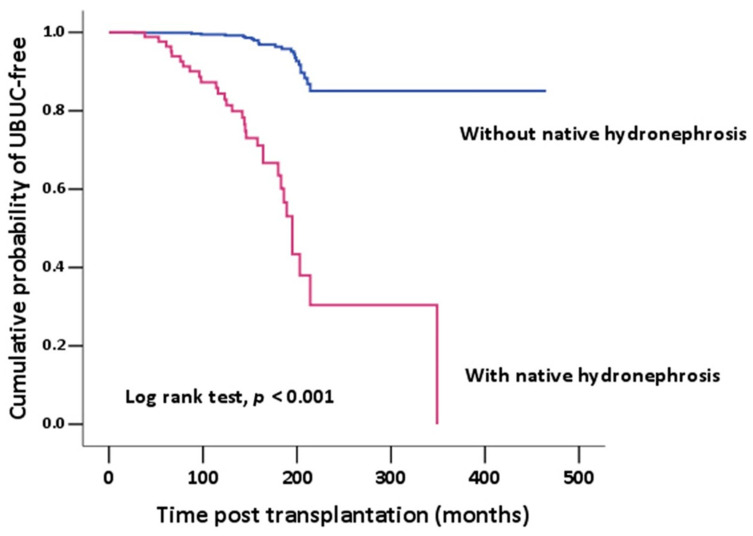
Kaplan–Meier analysis of the cumulative probability of UBUC-free showed significant difference between the post-KT patients with or without native kidney hydronephrosis. UBUC-free was statistically significantly higher in those patients without native kidney hydronephrosis.

In Table 3, stepwise logistic regression analysis shows the predictive factors of de novo UBUC. Univariate analysis showed that both gender and native hydronephrosis are significant risk factors for UBUC (OR, 0.324; 95% CI, 0.197–0.593; *p* < 0.001, OR, 18.740; 95% CI, 10.319–34.034, *p* < 0.001, respectively). However, multivariate analysis only showed native hydronephrosis as a risk factor that had a statistically significant correlation with post-KT UBUC (OR, 16.033; 95% CI, 8.660–29.683; *p* < 0.001). We further obtained a potential trend of native hydronephrosis for UBUC using the Kaplan–Meier method with log rank test, *p* values < 0.001 (Figure 1).

Further, we demonstrated the tumor characteristics of 42 post-KT UBUC patients (16 patients with UTUC before UBUC were excluded) with or without native hydronephrosis (Table 4). High-grade UBUC was observed in our post-KT cohort; however, no differences were found between the two groups (91.3% with hydronephrosis versus 68.4% without hydronephrosis, *p* = 0.649). Moreover, there was no significant difference in the T stage between the two groups. The number of multiple bladder tumor lesions upon initial diagnosis was higher in the native hydronephrosis group (47.8% versus 15.8%, *p* = 0.028). Table 4 shows that the UBUC recurrence rate was higher than 40% during follow-up. In addition, synchronous UTUC was observed to be more frequent in the native hydronephrosis group (65.2% versus 21.1%, *p* = 0.004).

## 4. Discussion

Unlike in Western countries, UC is the most common post-transplantation malignancy in Taiwan. Besides ethnicity, the customs, the environmental background of using herbs as traditional Chinese medicine, and arsenic-contaminated groundwater intake may partially explain the extraordinarily high prevalence of UC in post-KT recipients in Taiwan [2,6]. Our institution is located in Central Taiwan and is one of the largest post-KT patient care centers in our country. During the 15-year study period, more than 1000 post-KT cases were followed up at our institution, and 58 post-KT patients were initially diagnosed with UBUC. Ou et al. reported an incidence (0.89%) of UC among 1910 uremic patients undergoing maintenance dialysis in Southern Taiwan [15]. While reviewing our data, we observed that a de novo UBUC incidence of 5.8% in post-KT recipients is a noteworthy issue in post-KT care and surveillance. The aggressiveness of UC in post-KT patients has been repeatedly published in the literature according to their immunosuppressive backgrounds [16]. Thus, the early detection of UC in this cohort is extremely important for patients and clinical physicians.

Estimates for the prevalence of BK virus infection in KT recipients range from 30% to 40%, which is similar to the results presented in Table 1 [17,18]. Even the BK virus usually causes an asymptomatic infection and remains dormant in the urothelium, and several studies have found that KT recipients with a positive case of BK virus face a relative risk of developing UC [11,19,20]. However, the difference found in BK virus infection prevalence between the groups in this study is not a discrepancy. Compared with the abovementioned studies, we defined BK virus infection only with a serologic test in viral load. A biopsy proved that polyomavirus-associated nephropathy and the decoy cell in urine cytology should be accounted for BK virus infection, which might have caused the bias in our findings. The BK virus oncogenic mechanism in UBUC in KT recipients arguably occurs through the inhibition of p53 and pRB, similar to HPV-associated viral oncogenic pathways [21]. However, the role of HPV in the etiology of UC remains debatable. One retrospective study observed 60% UC to be positive for HPV-16, and another study found that high HPV-16 and 18 DNA detection rates in bladder tissue biopsy are significantly associated with UBUC [22,23]. The recent meta-analysis, which included 26 studies, found no significant association between HPV and bladder cancer, despite the relatively high prevalence of the virus [24].

For post-KT patients, there is a two- to four-fold risk of developing and dying from cancer compared with the general population. In addition, sex disparities in cancer incidence and mortality after KT have also been assessed [25]. Male KT recipients have been observed to have a 20–30% higher risk of de novo cancer [26]. However, females have also been observed to have a higher risk of developing selected malignancies after KT [2,27,28]. Generally, males are more likely to have UBUC than females; however, we found female predominance in our post-KT cohort (Table 2). In our previous study, female and native hydronephrosis were found to be statistically significant risk factors for de novo UTUC after KT [14]. Our current initial UBUC study was discordant with previous reports; our multivariable logistic regression analysis found no significant difference in terms of sex (Table 3). Another previous study from Taiwan showed no significant association between sex and UBUC survival outcomes after adjusting the risk factors for the general population; however, the role of immunosuppressed populations, such as post-KT patients, in the survival of UC is not adequately clear [29]. Malignancy originating from a graft kidney is rare; thus, it was expected that there was no significant difference in the graft hydronephrosis panel (Table 2). The mean duration from kidney transplantation to the diagnosis of bladder cancer was 7.0 ± 5.1 years, which is in line with previous studies [16,30,31,32]. In addition, we found that older age at KT might be a risk factor for UC, which is also in accordance with past publications [6].

Routine cytology and sonography examinations are basic in the post-KT follow-up protocol, and cystoscopy is the standard subsequent examination for hydronephrosis and hematuria in post-KT recipients. Cystoscopy with biopsy is also crucial for the initial detection and tissue proof of UBUC. Short term repeated cystoscopies can cause discomfort for patients and are one of the reasons for the high cost of UBUC care expenditure. While urine cytology is a currently and widely used urinary biomarker, its sensitivity in low-grade tumors is poor. Recently, urinary biomarker tests (UBTs) have been used as potential alternatives or adjuncts to establish the possible diagnosis of UBUC or disease recurrence in order to decrease uncomfortable and unnecessary cystoscopies. Food and Drug Administration (FDA) and European Medicines Agency (EMA) have approved NMP 22, BTA, UroVysion, and ImmunoCyt/uCyt+ for UBUC detection. However, a meta-analysis, which enrolled 57 studies, disclosed that these UBTs could miss 18 to 43% of patients with positive UBUC and yield false-positive results in 12 to 26% of patients who were negative of UBUC. Combining urinary biomarkers with cytology increases sensitivity, but 10% of positive cases may still be missed [33]. Another meta-analysis of 21 studies including 7330 patients showed that new developed UBTs with promising diagnostic values and demonstrated with sensitivities up to 93%, specificities up to 84%, with positive predictive values up to 67%, and negative predictive values up to 99% in the NMIBC. Furthermore, these innovative UBTs prevented unnecessary cystoscopy with up to 740 procedures during the follow-up of 1000 patients [34]. These novel UBTs include Xpert bladder cancer (Cepheid; Sunnyvale, CA, USA), Uromonitor (U-Monitor; Porto, Portugal), Cxbladder Monitor, and Triage and Detect (Pacific Edge; Dunedin, New Zealand) and have become commercially available for surveillance and early detection of UBUC. However, although novel UBTs have shown promising results, to date, guidelines still do not recommend routine use of any UBTs [35].

In the last few decades, the overall prognosis and patient survival for UBUC have remained unsatisfactory. Several tissue-based molecular biomarkers have emerged as potential rising stars. In one Taiwanese study, the researchers focused on the influence of the JNK cascade on UC progression. JNK-associated signaling pathways modulate tumor metabolic reprogramming, proliferation, and migration. The receptor tyrosine kinase-like orphan receptor 2 (ROR2) is the most expressively upregulated gene during UBUC progression. High ROR2 expression usually leads to tumor aggressiveness and poor prognosis [10]. Another tissue-based molecular biomarker developed in recent studies is serine proteinase inhibitor clade E member 2 (SERPINE2), which is a member of the serine protease inhibitor superfamily. SERPINE2 may also play an important role in activating and rousing immune cells, which is significantly associated with tumor treatment response. Increased SERPINE2 expression is identified as an adverse pathological feature association and an independent prognostic factor of overall survival [36]. Human epidermal growth factor-containing fibulin-like extracellular matrix protein 1 (EFEMP1) is a secreted extracellular matrix glycoprotein and an important component of basement membranes and extracellular matrix, which is broadly expressed during body development and in adult tissue. Abnormalities of this gene may strengthen the capacity of tumor cell invasion and metastasis [37]. Integrating these tissue-based biomarkers into clinical decision-making and risk stratification with standard pathologic predictors may be beneficial in UBUC care. However, further validation may be necessary to verify the underlying molecular mechanisms before clinical application.

As previously mentioned, the available methods for detecting de novo UBUC in post- KT recipients have been limited. Cystoscopy has traditionally been the primary approach for identifying tumors of the urinary bladder, but it can cause discomfort for the patient and can be costly. Periodic renal sonography is a useful imaging modality in the KT follow-up protocol as it is a safe, non-invasive technique that does not expose the patient to ionizing radiation. Without physiologic urine flush, native ureter stricture is commonly observed in nonfunctioning kidneys and post-KT recipients. Another contributing factor in the intravesical obstruction of ureteral orifices could be UBUC lesions [38]. Hydronephrosis in UTUC is associated with advanced disease and worse oncological outcomes, and it may also predict advanced disease and positive surgical margin in UBUC after cystectomy [39,40,41]. Our previous study disclosed that native hydronephrosis may predict subsequent UTUC in post-KT patients [14]. However, to date, there has been no related research on the association between native hydronephrosis and UBUC in post-KT patients. In the current study, post-KT patients with native hydronephrosis clinically presented with multiple bladder tumors and UBUC with subsequent UTUC (Table 4). Unlike hydronephrosis in advanced UBUC without UTUC caused by tumor infiltration around the ureters or lymph node external compression, the mechanism of hydronephrosis in our study group seems to be multiple bladder lesions in long-term disuse bladder, which led to ureteral obstruction. Impaired and even non-renal function demonstrated in native kidneys, speculatively, can be more than an obvious result of urine flow disturbance. Accompanied with narrowing stricture native ureters, even early-stage UBUC in post-KT patients may also cause hydronephrosis. The limitations of Bacillus Calmette-Guérin (BCG) instillation and immunotherapy after operation in post-KT patients lead to much more recurrent and progressive disease in comparison with UBUC patients without KT. According to the highly synchronous UBUC and UTUC and multifocal bladder tumor characteristics, sharing decision-making paradigms with patients about their treatment are warranted. These treatment options include the transurethral resection of the bladder tumor (TURBt) and the concurrent diagnostic ureterorenoscopy for upper tract work-up, may be mandatory for post-KT patients with native hydronephrosis.

This retrospective study has several limitations. First, because this is a retrospective cohort study, selection bias may be inevitable. The results from a single-center study may not be representative and generalized to other populations or races. Second, the shortage of urinalysis, cytology, and survival data may preclude their further analysis and interpretation. However, we collected more than 1000 cases during our 15-year study period, with a mean follow-up time of 118 months. The deviation of inference may be lessened. Third, immunosuppressive agent use is diverse based on different nephrologist preference, which makes analysis difficult. Fourth, there are no detailed bladder tumor location data; thus, ureteral orifice obstruction causing native hydronephrosis in long-term disuse bladder could not be proved. Fifth, the severity of kidney hydronephrosis was graded by images (sonogram and computed tomogram) using a routine protocol; the native hydronephrosis condition was only periodically traced during the complete follow-up protocol.

## 5. Conclusions

In conclusion, native hydronephrosis in post-KT recipients intimates de novo UBUC development and possible subsequent UTUC. Post-KT UBUC is characterized by pathologically aggressive and multiple foci lesions. Periodical renal sonography is crucial for post-KT care as it can detect early and subsequently monitor hydronephrosis, which could possibly lead to the development of UBUC in post-KT patients. 

## Figures and Tables

**Table 1:** Characteristics of KT patients.

Characteristic	Total Population n = 1005
Transplantation age, y/o	48.2 ± 12.0
Gender	
Male	612 (60.9%)
Female	393 (39.1%)
Smoking history	168 (22.7%)
Hypertension	648 (64.5%)
Diabetes mellitus	541 (53.8%)
Dyslipidemia	820 (82.0%)
Hyperuricemia	780 (78.0%)
Hepatitis B virus infection	172 (31.3%)
Hepatitis C virus infection	94 (19.1%)
BK virus infection,	281 (39.0%)
Native Hydronephrosis	88 (9.4%)
Unilateral	74 (84.1%)
Bilateral	14 (15.9%)
Graft Hydronephrosis	217 (23.1%)
Average time from KT to UBUC, years	7.0 ± 5.1

KT, kidney transplantation; UBUC, urinary bladder urothelial carcinoma.

**Table 2:** Characteristics of KT patients with or without UBUC.

Characteristics	With UBUC n = 58	Without UBUC n = 947	*p* Value
Transplantation age, y/o	51.6 ± 9.5	48.0 ± 12.0	0.029
Gender			<0.001
Male	21 (36.2%)	591 (62.4%)	
Female	37 (63.8%)	356 (37.6%)	
Smoking history	6 (12.2%)	162 (23.5%)	0.007
Hypertension	36 (62.1%)	612 (64.6%)	0.693
Diabetes mellitus	32 (55.2%)	509 (53.7%)	0.833
Dyslipidemia	51 (87.9%)	769 (81.6%)	0.226
Hyperuricemia	44 (75.9%)	736 (78.1)	0.685
Hepatitis B virus infection	9 (28.1%)	163 (31.5)	0.687
Hepatitis C virus infection	4 (15.4%)	90 (19.3%)	0.620
BK virus infection	16 (45.7%)	265 (38.6%)	0.402
Native Hydronephrosis	31 (56.4%)	57 (6.4%)	<0.001
Unilateral	29 (93.5%)	45 (78.9%)	
Bilateral	2 (6.5%)	12 (21.1%)	
Graft Hydronephrosis	12 (21.8%)	205 (23.2%)	0.811

KT, kidney transplantation; UBUC, urinary bladder urothelial carcinoma.

**Table 3:** Stepwise logistic regression analysis for the predictive factors of de novo UBUC in post-KT patients.

Clinical Variable	Univariable			Multivariable		
	OR	95% CI	P	OR	95% CI	P
Age > 65	1.029	0.361–2.939	0.957			
Gender (M/F)	0.324	0.197–0.593	<0.001	0.563	0.301–1.050	0.071
Smoking history	0.455	0.190–1.088	0.077			
Hypertension	0.896	0.518–1.548	0.693			
Diabetes mellitus	1.059	0.622–1.805	0.833			
Dyslipidemia	1.639	0.731–3.674	0.230			
Hyperuricemia	0.880	0.473–1.637	0.686			
HBV	0.850	0.385–1.877	0.687			
HCV	0.760	0.255–2.259	0.621			
BK virus	1.338	0.676–2.648	0.403			
Native hydronephrosis	18.740	10.319–34.034	<0.001	16.033	8.660–29.683	<0.001
Graft hydronephrosis	0.923	0.478–1.783	0.811			

OR, odds ratio; CI, confidence interval.

**Table 4:** Tumor characteristics in KT patients with UBUC.

Characteristics	With Native Hydronephrosis n = 23	Without Native Hydronephrosis n = 19	*p* Value
Histologic grade			
Grade I, Low grade	2 (8.7%)	2 (10.5%)	0.649
Grade II, III, High grade	21 (91.3%)	13 (68.4%)	0.649
Unknown	0 (0%)	4 (21.1%)	
T stage			
Ta	4 (17.4%)	2 (10.5%)	0.815
Tis	1 (4.3%)	1 (5.3%)	0.706
T1	12 (52.2%)	5 (26.3%)	0.314
T2	3 (13.0%)	4 (21.1%)	0.225
T3	0	0	
T4	0	0	
Unknown	3 (13.0%)	7 (36.8%)	
Multiple bladder tumors on initial	11 (47.8%)	3 (15.8%)	0.028
Bladder recurrence	13 (56.5%)	9 (47.4%)	0.554
Synchronous UTUC	15 (65.2%)	4 (21.1%)	0.004
UBUC first	3 (13.0%)	1 (5.3%)	0.393

KT, kidney transplantation; UBUC, urinary bladder urothelial carcinoma.

## Data Availability

The data presented in this study are available on request from the corresponding author with a reasonable reason.
